# Age- and sex-related development of osteosarcopenia in the aging *Octodon degus* rodent model

**DOI:** 10.3389/fragi.2025.1486670

**Published:** 2025-02-13

**Authors:** Pablo Gallo-Soljancic, Maria Egle De Stefano, Ana-María Lucas-Ochoa, Consuelo Sánchez-Rodrigo, Lorena Cuenca-Bermejo, Ana-María González-Cuello, Emiliano Fernández-Villalba, María-Trinidad Herrero

**Affiliations:** ^1^ Clinical & Experimental Neuroscience (NiCE), Department of Human Anatomy and Psychobiology, Institute for Aging Research, School of Medicine, European University for Well-Being (EUniWell), Campus Mare Nostrum, University of Murcia, Murcia, Spain; ^2^ Institute for Bio-Health Research of Murcia (IMIB-Pascual Parrilla), Campus of Health Sciences, El PalmarMurcia, Spain; ^3^ Department of Biology and Biotechnologies “Charles Darwin”, Sapienza University of Rome, Rome, Italy; ^4^ Center for research in Neurobiology “Daniel Bovet”, Sapienza University of Rome, Rome, Italy

**Keywords:** aging, osteoporosis, sarcopenia, *Octodon degus*, osteosarcopenia, sex

## Abstract

The increase in life expectancy in recent years has resulted in a higher incidence of age-related diseases. Among these, osteoporosis and sarcopenia, collectively known as osteosarcopenia, have the most significant impact on the quality of life, general health and frailty in the elderly. As for other age-related diseases, pre-clinical studies on these conditions are primarily limited by the availability of experimental model systems. The *Octodon degus* (*O. degus*) is a long-lived diurnal rodent identified as a potential tool in ageing research. However, age-related osteosarcopenia changes have not yet been explored. In this study, male and female *O. degus* from juvenile to senile ages were used (6 months–7 years old). Changes in the volume of several forelimbs and hindlimbs muscles, e.g., biceps femoris, triceps brachii, femur, and humerus, were evaluated using computed tomography. Aged animals showed a significant decrease in muscle volume in both hindlimbs and forelimbs, along with a significant reduction in cortical bone volume. With ageing, sex differences were also observed, with female *O. degus* showing greater cortical bone volume in both hind and forelimbs, and greater muscle mass in the sole hindlimbs, compared to male. These findings enhance the characterization of *O. degus* as a model to study age-related pathologies, also considering sex differences, and lay down solid foundations for future studies that can address in more detail the molecular mechanisms underlying the initiation and progression of osteosarcopenia.

## 1 Introduction

As life expectancy increases, the elderly population grows (Sharples et al., 2015), leading to a rise in age-related diseases like osteoporosis and sarcopenia (Krut’ko et al., 2018). These conditions frequently co-occur, resulting in the geriatric syndrome known as osteosarcopenia (Hirschfeld et al., 2017; Kirk and Al Saedi, 2019; Kirk et al., 2020; Fagundes Belchior et al., 2020; Clynes et al., 2021), which affetcs 5%–37% of individuals over 65. This syndrome significantly impacts quality of life and poses a considerable socioeconomic burden, particularly in countries with aging populations (Hassan and Duque, 2017; Hirschfeld et al., 2017; Kirk and Al Saedi, 2019; Kirk et al., 2020).

Osteoporosis is a metabolic bone disease characterized by imbalanced bone remodeling (Kenkre and Bassett, 2018), leading to the deterioration of bone microarchitecture. Between the ages of 30 and 70, approximately 30% of bone mass is lost (Clynes et al., 2020), with women being more affected than men (Melton, 2001; Krut’ko et al., 2018). The decline in estrogen after menopause plays a crucial role in this loss, as it correlates with decreased bone density and increased inflammation, which further contributes to osteoporosis (Richelson et al., 1984; He et al., 2016; Wu et al., 2021). The gut microbiota also influences bone health, as studies show that restoring the microbiota in germ-free mice can normalize bone mass (Ohlsson et al., 2014; Britton et al., 2014). Osteoporosis significantly raises the risk of fractures and related complications (Tournadre et al., 2019), resulting in substantial healthcare costs (Clynes et al., 2020). In Europe alone, the estimated cost was 37 billion euros in 2010, with projections indicating a 25% increase by 2025 (Hernlund et al., 2013; Ethgen et al., 2017).

Sarcopenia, the progressive loss of skeletal muscle mass, strength, and function, is another critical issue in the elderly (Frontera et al., 1991; Metter et al., 1999). It leads to disabilities, cognitive decline, and higher mortality rates (Liguori et al., 2018; Farmer et al., 2019). Sarcopenia is caused by multiple factors, including neurological issues (e.g., loss of motor neurons), hormonal changes (e.g., decreased testosterone), poor nutrition, and inactivity (Wozniak and Anderson, 2009; Cruz-Jentoft et al., 2019). Muscle loss begins around age 50, with individuals losing 1%–2% of muscle mass per year, and by age 80, 40%–50% of people are affected (Sharples et al., 2015). However, the underlying molecular mechanisms of sarcopenia remain poorly understood (Pascual-Fernández et al., 2020).

Currently, there are no precise methods to directly measure bone strength. Bone mineral density (BMD) is commonly used as an indirect measure, accounting for about 70% of bone strength. Computed tomography (CT) is a reliable method for assessing BMD (Klibanski et al., 2001; Singer, 2006; Cruz-Jentoft et al., 2019), and along with magnetic resonance imaging (MRI), is also used to evaluate muscle mass. CT’s widespread availability and non-invasive nature make it a popular choice in healthcare settings (Maravilla and Murry, 1981; Ko et al., 2022).

To better understand and develop treatments for osteosarcopenia, accurate studies on its molecular mechanisms and aging progression are essential. The *Octodon degus* (*O. degus*), a diurnal rodent native to South America, has emerged as a promising animal model for such studies. With a life expectancy of 5–7 years and a circadian cycle similar to humans, *O. degus* naturally develops age-related diseases like Alzheimer’s, type II diabetes, and kidney alterations (Inestrosa et al., 2005; Tarragon et al., 2013; Cuenca-Bermejo et al., 2020). This rodent has proven versatile in various research areas, including sleep deprivation and memory enhancement (Tarragon et al., 2014; Estrada et al., 2015a; Estrada et al., 2015b; Estrada et al., 2019). The primary goal of this study using *O. degus* has been to characterize the progressive bone and muscle changes during aging, with a particular focus on sex differences.

## 2 Materials and methods

### 2.1 Ethical statement

Experimental procedures followed the regulations indicated in the experimental guidelines, and the procedures complied with the European Community Council Directive (2010/63/UE) and the Ethics Committee of the University of Murcia (project number: A13170102/CEEA-OH AMP/MOD 103/2014 + 2018). The “3 R’s principle” was applied throughout the study, and the investigators made significant efforts to minimize the procedure’s impact on animals.

### 2.2 Animals

Ninety-six healthy *O. degus* were used in this study. The animals were randomly assigned to experimental groups based on sex (female or male) and age: i) 6 months old (juvenile); ii) 1 year old (adult); iii) 4–5 years old (old); and iv) 6–7 years old (senile). *O. degus* were sourced from our colony in the animal house of the Biomedical Research institute of Murcia (IMIB, University of Murcia). Two to four animals from the same experimental group were housed in plexiglass cages with wood shavings and environmental enrichment. Cages were cleaned weekly, and the animals were housed in a room with controlled temperature (23°C ± 1°C) and humidity (55% ± 5%), with *ad libitum* access to food and water. A 12:12 light/dark cycle (light from 8:00 to 20:00 h) was maintained using an electronic timer (DataMicro, Orbis). The health status of the animals was monitored by the veterinarian of the center. Table 1 lists the animals used, divided by age and sex; average weights ± Standard Deviations are also indicated.

**TABLE 1:** *O. degus* were divided into four age-related groups. Male and females were analyzed separately. Weight in grams ± Standard Deviation and number of animals for each subgroup is indicated.

Groups	Age (year)	Sex	Weight (grams)	Number
Juvenile	0.5	Male	210.56 ± 12.84	6
		Female	184.28 ± 24.92	9
Adult	1	Male	217.71 ± 13.05	10
		Female	194.84 ± 8.63	11
Old	4–5	Male	283.00 ± 18.89	16
		Female	244.80 ± 5.93	18
Senile	6–7	Male	269.53 ± 20.39	16
		Female	223.93 ± 17.67	10

### 2.3 Computerized tomography

**FIGURE 1:**
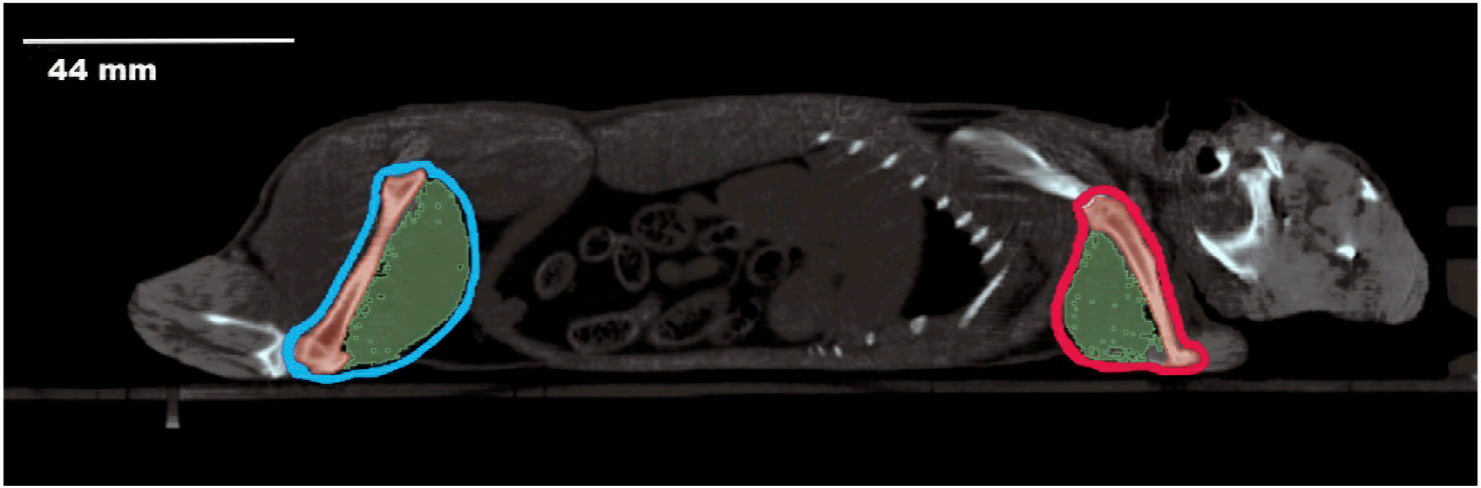
Sagittal Computerized Tomography scan of an *O. degus*. The head is visible on the right side of the image, while the posterior part of the body is on the left. The hindlimbs, including femur and biceps femoris, are outlined in light blue. The forelimbs, comprising the humerus and triceps brachii, are outlined in red. Bone structures are depicted in dark red, and muscles are shown in green.

Computerized tomography (CT) was performed using the Albira CT/SPECT/PET 108 tri-modal preclinical scanner (Serial Number 0900126, Bunker Corporation, Massachusetts, United States). The scanner is composed of 8 detectors per ring, and 1 to 3 rings per system configured for Lutetium Orthosilicates Cristal (LSO), a patented system. To prevent artefacts related to movement during the scan, animals were anaesthetized with a mixture of 75 mg/kg of ketamine (Anesketin^®^ 100 mg/mL, Dechra Veterinary Products SLU, Barcelona) and 0.5 mg/kg of medetomidine (Domtor^®^1 mg/mL, Ecuphar^®^, Barcelona). The scanning conditions were set at 45 kV voltage and 400 μA current. An aluminum filter (0.5 mm) was used to harden the beam. The X-ray detector was a digital flat panel with 2,400 Å∼ 2,400 pixels and a field of view of 70 Å∼ 70 mm^2^. Animals were placed inside the bed in their natural position, and the whole body of the animal was scanned, from the mouth to the end of the tail (Figure 1). The complete scan was composed of sequential sections of 0.16 mm, and the total exposure per animal was 35–40 min. Images obtained were reconstructed by the algorithm of filtered back projection using the Albira Suite 5.0 Reconstructor software (Bruker^®^, Karksruhe, Germany), which enabled the acquisition of images in the three orthogonal planes (coronal, sagittal, transversal). The hindlimbs, comprising the femur and biceps femoris, and the forelimbs, comprising the humerus and triceps brachii, were the focus of our investigation. In both cases, the specific region of interest was identified, and the amount of tissue was determined based on the estimated threshold range (Table 2).

**TABLE 2:** Threshold range for *O. degus* tissues analyzed by computerized tomography.

Tissues	Minimum	Maximum
Muscle	0	170
Trabecular bone	200	400
Crtical bone	500	800

### 2.4 Muscle and bone analysis

Reconstructed images were analyzed using the PMOD 3.5 (PMOD Technologies Ltd., Switzerland) software package, AMIDE 1.0.4 (Amide a Medical Image Data Examiner) software package, and Amira 2019.1 software systems for 3D visualization (Zuse Institute Berlin (ZIB), Thermo Fisher Scientific). Contouring of both forelimbs and hindlimbs was manually performed for each animal with PMOD 3.5, delimiting the region of interest (ROI). To evaluate changes in bone and muscle content in the limbs, the volume of interest (VOI), measures as cubic centimeters (cm^3^), occupied by cortical bone, trabecular bone, and muscle were calculated. To this aim, the ROI of each limb was segmented using the specific density values (HU) for each component, as described in Table 2. In this study, we used the muscle assessment values reported in Figueiredo et al. (2018) and the bone assessment values reported in Barwick et al. (2017). Final values were determined using the AMIDE software. The quantitative analysis of each component was performed by comparing the relative volumes (expressed as percentage, %) to the total volume of the limb. Once the volume was obtained, a segmentation *per* range of threshold was performed to get the volume of cortical bone, trabecular bone, and muscle (Table 2). Percentage for each tissue was calculated distinguishing hindlimbs and forelimbs to obtain the average for each animal. The Amira 2019.1 software was used to highlight the analyzed areas and create the 3D images.

### 2.5 Statistical analysis

Values are expressed as the mean ± standard deviation (SD). To determine the effect of sex and age on bone and muscle volumes, Two-Way ANOVA analysis followed by Tukey’s multiple comparison test and the normality test were applied. Differences were considered statistically significant at p ≤ 0.05. All statistical analyses were performed using the GraphPad Prism 8.0 software package (GraphPad Software, San Diego, California).

## 3 Results

### 3.1 Age and sex-related variations in muscle mass volume

**FIGURE 2:**
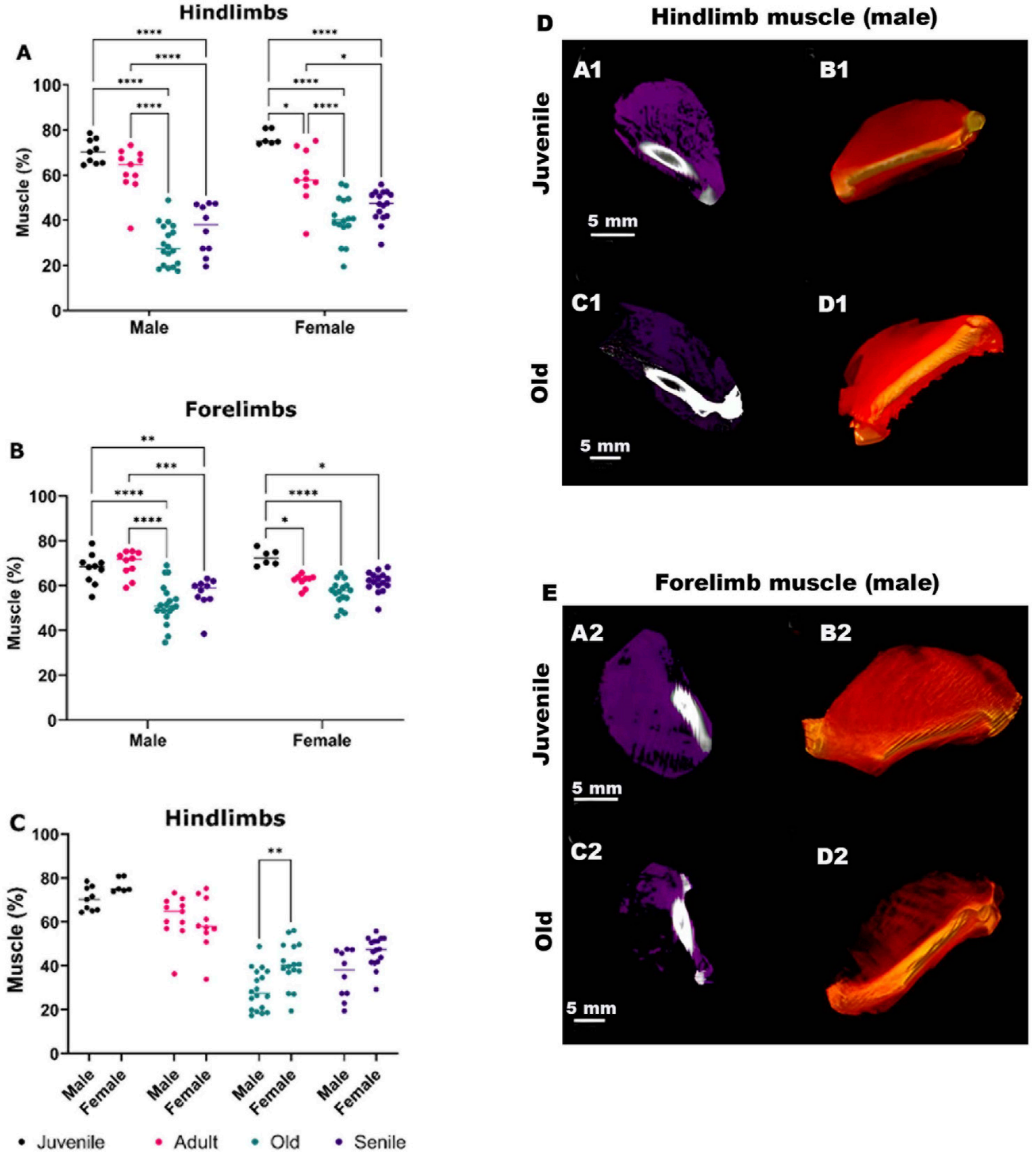
Quantitative sex-specific analysis of hindlimb and forelimb muscle mass volume in *O. degus* obtained by computerized tomography (CT), along with representative CT images. **(A)** Muscle mass in hindlimbs, **(B)** muscle mass in forelimbs and **(C)** comparison of hindlimb muscle mass between sexes. Muscle mass is expressed as a percentage of total volume, calculated by delineating the area of each muscle. *p ≤ 0.05, **p ≤ 0.01, ***p ≤ 0.001, ****p ≤ 0.0001. **(D, E)** Sagittal views of representative CT images of hindlimb **(D)** and forelimbs **(E)** muscles from juvenile and old *O. degus*. In panels (A1,C1,A2,C2), muscle tissues are depicted in purple, while in panels. (B1,D1,B2,D2) 3D renderings display muscle tissues in red.

In the initial analysis, we quantified changes in the percentage of muscle mass volume with aging, from juvenile to senile stages, in both the hindlimbs and forelimbs of male and female *O. Degus* (Figure 2). In the hindlimbs (Figure 2A), a significant decrease in the percentage of muscle mass with age was observed in both male and female. Specifically, in males there was no significant difference in muscle mass between the juvenile and adult groups, although a tendency for muscle volume reduction was already noticeable. A drastic decrease compared to juvenile and adult animals was observed when rodents reached the old stage of their life, with muscle mass volume stabilizing in senile *O. Degus*. Conversely, females exhibited a slight, but significant, reduction in muscle mass as early as the adult stage compared to juveniles. This loss further increased in old and senile *O. Degus*, with muscle mass volume remaining stable between these two age groups. Figure 2D depicts representative CT images of juvenile and old *O. Degus*, showing the measured reduction in hindlimb muscle mass volume.

The forelimbs followed a similar trend as the hindlimbs (Figure 2B), though the decrease in the mean percentage of the muscle mass was less pronounced compared to the hindlimbs. In females, the decrease in muscle mass was observed between juvenile *O. Degus* and all the other age group, but no further muscle loss was evident between the adult, old, and senile groups. Figure 2E, shows representative CT images of juvenile and old *O. Degus*, showing the measured reduction in the forelimb muscle mass volume.

Finally, comparing age-matched males and females (Figure 2C) revealed a significant difference in old animals, with males showing a higher loss of muscle mass volume compared to females. This indicates a gender difference in muscle composition at this age.

### 3.2 Age and sex-related variations in bone mass volume

**FIGURE 3:**
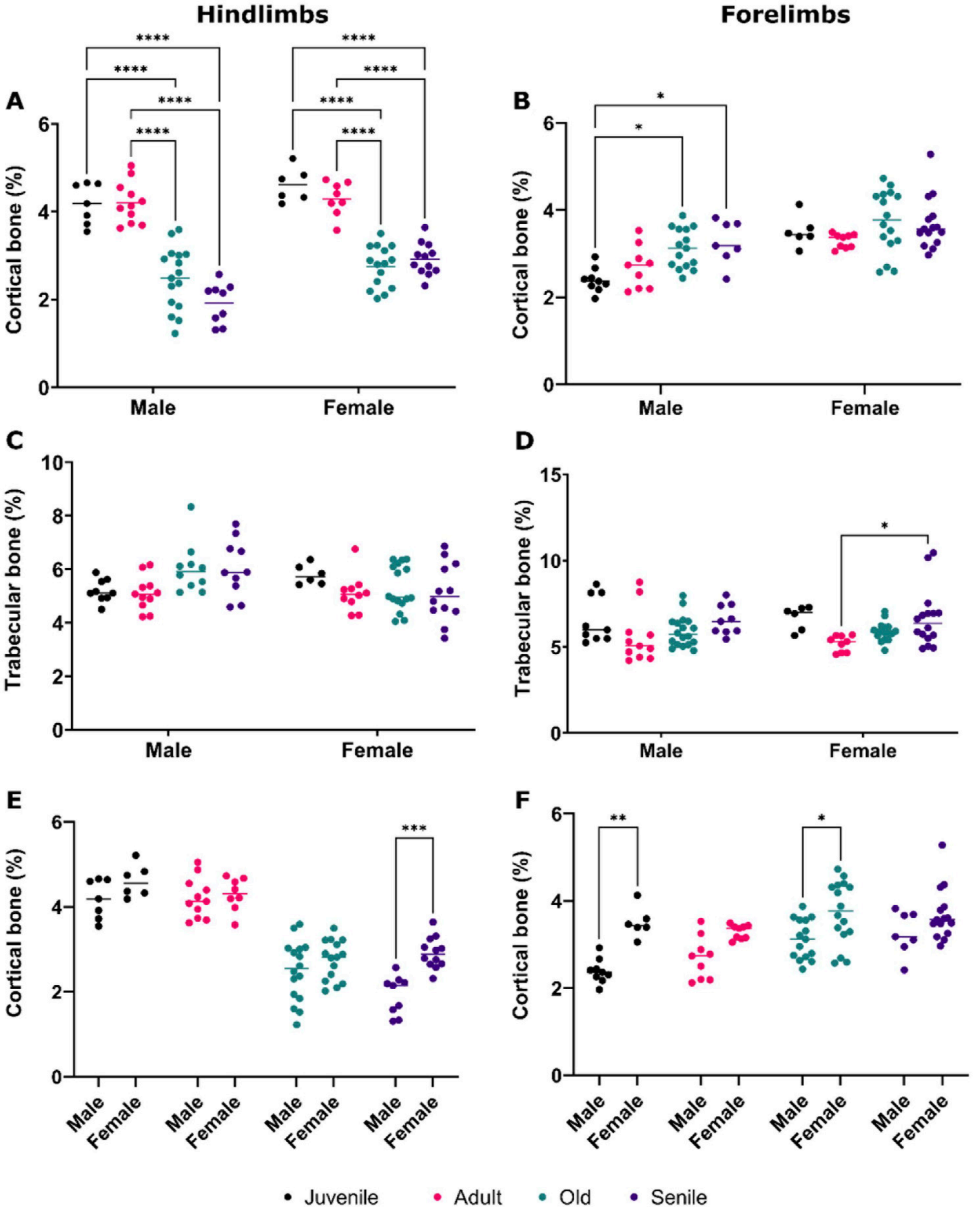
Comparative quantitative analysis of bone volume in the hindlimbs and forelimbs of male and female *O. degus,* categorized by age groups. **(A)** Cortical bone volume and **(C)** trabecular bone volume in the hindlimbs. **(B)** Cortical bone volume and **(D)** trabecular bone volume in the forelimbs. **(E)** Comparison of cortical bone volume hindlimb between sexes, and **(F)** comparison of cortical bone volume in the forelimbs between sexes. Bone volume is expressed as a percentage of total volume, calculated by delineating the area where the bone is located. *p ≤ 0.05, **p ≤ 0.01, ***p ≤ 0.001, ****p ≤ 0.0001.

A comparative quantitative analysis of bone volume was performed for both cortical and trabecular bones in the hindlimbs and forelimbs of male and female *O. degus*. In the hindlimbs, both males and females showed a significant decrease in the percentage of cortical bone volume, following a similar trend (Figure 3A). Notably, as previously observed in male muscles, no differences were found between juvenile and adult animals of either sex, with a marked decrease in bone volume percentage evident in the older groups. Senile male and female *O. Degus* did not experience further bone loss compared to old animals. In the forelimbs (Figure 3B), only males exhibited a tendency to increase the percentage of cortical bone volume with age, reversing the pattern observed in the hindimbs. Differences, however, were statistically significant only between male juvenile *O. degus* and both old and senile animals. In contrast, no significant differences were observed across any of the age groups in females.

The analysis of trabecular bone volume in the hindlimbs (Figure 3C) did not yield statistically significant results, in either males or females. In contrast, a slight, but significant increase in bone volume was measured in the forelimbs between juvenile and senile female *O. degus* (Figure 3D).

**FIGURE 4:**
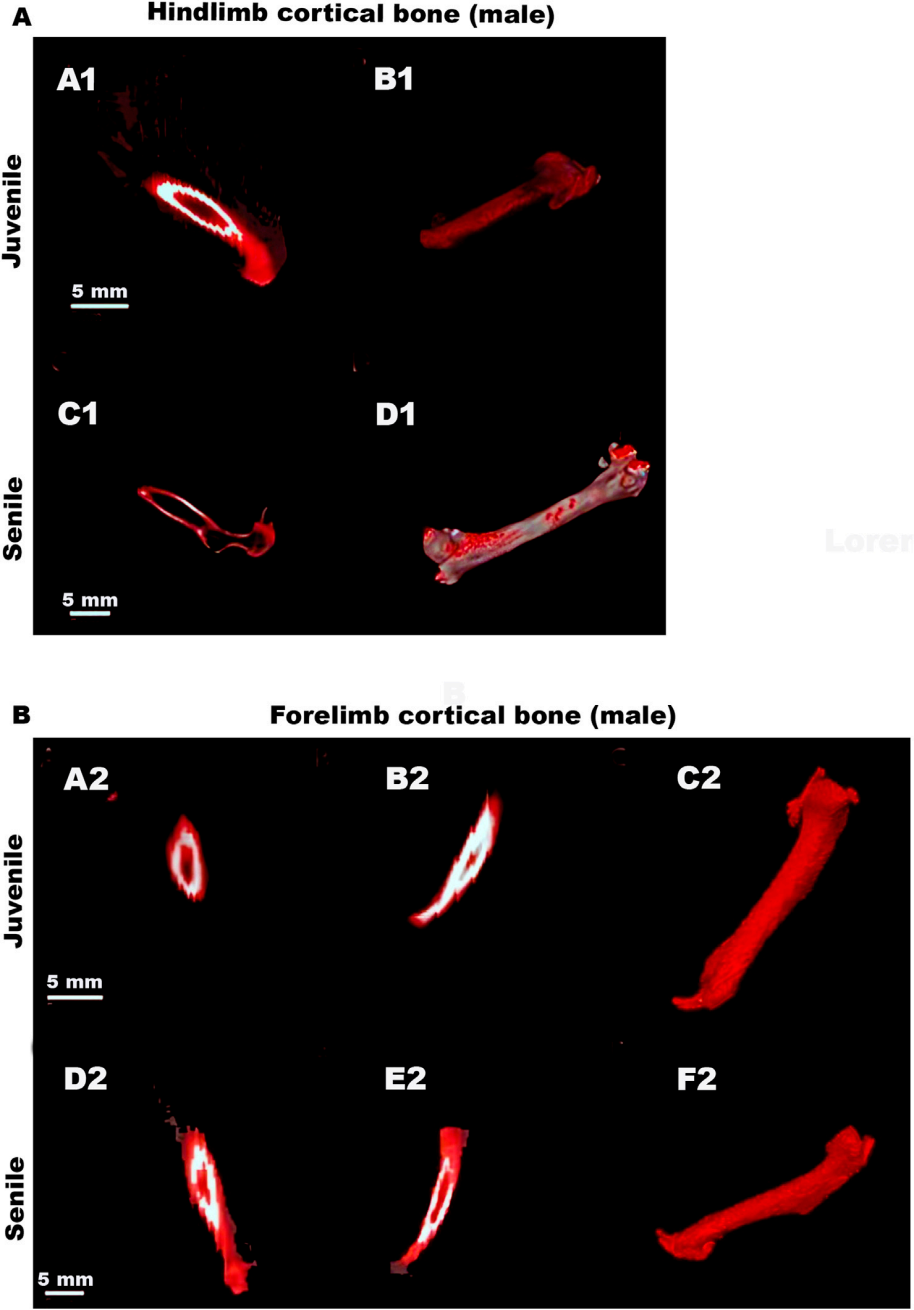
Representative CT images of hindlimbs and forelimb cortical bones in male *O. degus*. Male hindlimb **(A)** and forelimb **(B)** cortical bones. (A1,C1) Sagittal views of the hindlimb cortical bones (depicted in red) in juvenile and senile male *O. degus*. (B1,D1) 3D renderings display bone tissue in red. (A2,D2) Coronal and (B2,E2) sagittal views of the forelimbs cortical bones (depicted in red) of juvenile and senile male *O. degus*. (C2,F2) Sagittal 3D renderings display bone tissue in red.

Figures 4A,B depicts representative CT images that clearly illustrate the reported decrease in cortical bone volume between the hindlimbs of juvenile and senile male *O. Degus*, as well as the increase observed in the forelimbs between juvenile and senile male animals.

Sexual dimorphism in cortical bone volume was noted in the hindlimbs of senile animals (Figure 3E), and in the forelimbs of juvenile and old *O. degus* (Figure 3F), with female bones consistently showing significant hypertrophy compared to males. This finding underscores a gender difference in bone composition.

### 3.3 Comparison of hindlimbs *versus* forelimbs according to sex

In a final analysis, we compared the volumes of cortical and trabecular bone, as well as those of muscle mass, between the hindlimbs and forelimbs within each age group of male and female *O. degus*.

**FIGURE 5:**
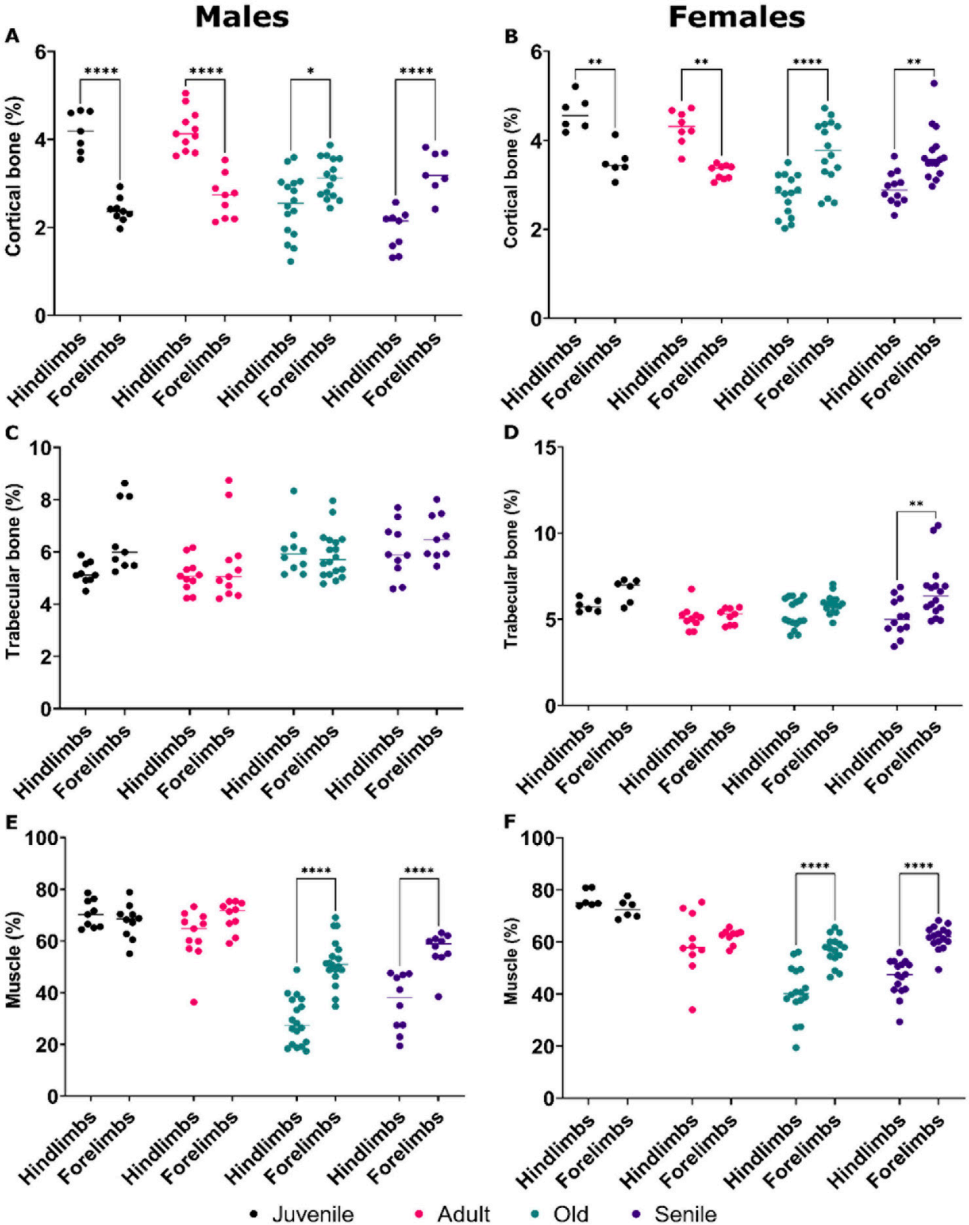
Comparative quantitative analyses of bone volume and muscle mass in the hindlimbs *versus* forelimbs of male and female *O. degus,* categorized by age groups. Comparisons are made between hindlimbs and forelimbs within the same age group. Percentage of cortical bone volume in **(A)** males and **(B)** females, percentage of trabecular bone volume in **(C)** males and **(D)** females, percentage of muscle mass volume in **(E)** males and **(F)** females. *p ≤ 0.05, **p ≤ 0.01, ****p ≤ 0.0001.

In juvenile and adult *O. degus*, the cortical bone volume of the hindlimb were consistently and significantly higher than that of the forelimbs, in both sexes (Figures 5A,B). However, with advancing age, this trend reversed; in old and senile animals, the percentage of cortical bone volume in the hindlimbs became significantly lower compared to the corresponding forelimbs in both males and females (Figures 5A,B), with the hindlimbs becoming the more affected extremity. No significant differences in trabecular bone volumes were observed between the hindlimb and forelimbs in both males and females *O. degus* (Figures 5C,D), except in senile females, where hindlimbs tissue volume was significantly lower than that forelimbs (Figure 5D), consistent with the cortical bone volume observations in the same group.

Regarding muscle mass, juvenile and adult *O. degus* of both sexes exhibited similar tissue volumes between the hindlimbs and forelimbs (Figures 5E,F). However, in old and senile animals of both sexes, the hindlimbs showed a lower percentage of muscle mass compared to the forelimbs (Figures 5E,F), reflecting the pattern observed for cortical bones in both genders and trabecular bones in females.

In Supplementary Figure S1 are shown couples of representative hindlimb CT images for each age group and sexes. In one of the two images, the ROI, within which bone density and muscle mass volumes were analyzed, is highlighted. These images are for illustrative purposes only, as all analyses were performed on reconstructed CT slices as detailed in Materials and Methods.

## 4 Discussion

In this study, we employed quantitative CT imaging to investigate the effect of progressive aging on muscle mass and bone volumes. The research was conducted using the aging animal model of *O. Degus* (Tarragon et al., 2013; Cuenca-Bermejo et al., 2020), with a focus on the muscles of both hindlimbs and forelimbs, while also considering sex differences.

Our finding revealed a significant age-related decline in muscle mass in both male and female limbs, with hindlimbs being more severely affected than forelimbs in older animals. Notably, the reduction in muscle mass percentage in the hindlimbs of old males was greater than in females, indicating a higher vulnerability in males to age-related muscles deterioration. However, at earlier ages (juvenile vs. adult), the females exhibit a slight but significant decline in muscle mass in both limbs. This might be attributed to the higher baseline testosterone levels in males, which are anabolic and promote muscle protein synthesis in both humans and rodents (Bhasin et al., 1996; Axell et al., 2006; Wang et al., 2000), potentially delaying the onset of muscle loss. Alternative, but not exclusive, is also the hypothesis of the beginning of a menopausal stage in adult females, as also discussed later on. The decay in muscle mass in old and senile male and female *O. degus* can be advocated to different factors, as also discussed later on, but hormones do play a relevant role. Existing research on female *O. degus* has focused primarily on estrogen and progesterone levels during the estrous cycle (Labyak and Lee, 1995; Mahoney et al., 2011) and, to our knowledge, no studies have fully and methodically investigated estrogen fluctuations during aging in this species. This leads to the assumption that menopause transition, which occurs by 6–8 years of age in captivity, combines with decreased hormone levels, behavioral and physiological changes, and parallels human menopause (Oliva et al., 2022), impacting muscle mass (Baltgalvis et al., 2010; Yoh et al., 2023). Our data suggest that this transition might begin even earlier (4–5 years). Similarly, reduction in androgen signaling in muscles determines reduced body and muscle mass, as well as reduced force production by fast-twitch muscles in male mice, as demonstrated *in vivo* on mice knockout for androgen receptors on fast-twitched fibers (MacLean et al., 2008; Hosoi et al., 2023). On a more general basis, the overall data indicate that fast-twitched muscles (type II), characteristics of hindlimbs, are those mainly susceptible to age-related decline, while slow-twitched muscles (type I), characterizing forelimbs, are less severely affected. This mirrors the changes observed in humans and rodents, where faster loss of motor units and fiber atrophy are seen primarily in fast-twitch muscle (Lang et al., 2010; Talbot and Maves, 2016), and regression analysis in humans has shown significantly higher muscle quality in the arms (∼30%) compared to the legs (Lynch et al., 1999). Furthermore, it is important to note that the effect of age and gender on muscle quality, as well as the extent of the changes observed, also depend heavily on the specific muscle group and the type of muscle action (concentric *versus* eccentric peak torque) used to assess strength (Lynch et al., 1999). A consistent observation between rodents and humans is that age-related declines in muscle strength, mass, and quality vary between the upper and lower limbs (de Lucena Alves et al., 2022a; de Lucena Alves et al., 2022b).

A similar trend to muscle mass decline was observed in cortical bone volume in the hindlimbs of older animals of both sexes, with a significant decrease in bone volume in older animals (old, senile) compared to juvenile and adult rodents. In contrast, the forelimbs displayed more heterogenous results: cortical bone volume in males increased significantly with age, while females showed no significant changes, although old and senile animals tended to exhibit an increase. This result, although unexpected, aligns with Wolf’s Law, which states that increased physical activity leads to greater mechanical loading, prompting the body to enhance cortical bone mass. Specifically, the more robust bone structure observed in the forelimbs compared to the hindlimbs of aging rodents can be attributed to their critical role in essential survival behaviors such as foraging, defense, and social interactions. The mechanical loads imposed by these activities, stimulate bone remodeling during aging and specifically enhance the cortical bone mass, compensating for the natural decline in muscle mass (Elissamburu and De Santis, 2011). Unlike cortical bone, trabecular bone volume remained largely unchanged across ages in both hindlimbs and forelimbs, except for a slight, but significant increase in the forelimbs of adult and senile females. These findings are particularly intriguing when considering the distinctions between cortical and trabecular bone. Cortical bone, which represent the compact and outer layer of long bones, is primarily responsible for providing strength and protection (Morgan et al., 2018). In contrast, trabecular (spongy) bone constitutes the inner layer and is characterized by a porous structure, which plays a crucial role in shock absorption and reducing bone weight (Morgan et al., 2018). The different result obtained for cortical and trabecular bones, especially related to aging male rodents, again aligns with the Wolf’s Law, according which the remodeling process, which allows the bone to structurally adapt to physical stress, occurs in the cortical bone rather than the trabecular one, aligning with the principles of bone mechanotransduction (Barak, 2024; Pearson and Lieberman, 2004; Leser et al., 2021). Overall, our results highlight an age-related decline in hindlimb cortical bone, which is crucial for maintaining strength in the more powerful limb during active stages of life, in both male and female *O. Degus*. However, in males, where certain behaviors, such as defense, and social interactions remain relatively active, cortical bone in the forelimbs increases significantly with age. While age-related changes have less impact on trabecular bone, the observed increase in the porous structure of the forelimb’s bones of senile female suggests a predisposition to developing “osteoporosis”. This condition is particularly prevalent in post-menopausal women, primarily due to the loss of estrogen (Albright et al., 1941). The mechanism though which this hormonal decline leads to bone deterioration are varied, including decreased calcium absorption, impaired vitamin D metabolism (Chevalley et al., 2022) and dysregulation of the adaptive immune system (Arron and Choi, 2000; Wu et al., 2021). Regarding sex differences in cortical bone, it is evidenced that males exhibit lower cortical bone density compared to females in both the hindlimbs of the senile group and the forelimbs of the juvenile and old groups. Although this difference is not statistically significant in other age groups, the observed trend suggests a constitutive difference in bone composition between male and female, which becomes more pronounced with age.

Age-related changes in muscle and cortical bone in *O. degu*s appear to occur in parallel. This observation aligns with the growing body of research supporting biochemical muscle-bone crosstalk (Li et al., 2019), and it further validates the use of *O. Degus* as a valuable model for studying the mechanisms underlying age-related musculoskeletal diseases, such as osteosarcopenia. Unlike other animal models, such as rats or mice with relatively short lifespans, or non-human primates with high maintenance costs (Komori, 2015; Oheim et al., 2016; Christian and Benian, 2020), *O. degus* naturally develop these conditions without the need for treatments or genetic modifications. Furthermore, our study underscores the importance of considering limb-specific variations in muscle and bone alterations related to age and sex within the context of osteosarcopenia. The presence of age-related sexual dimorphism in both muscle mass and cortical bone volume suggests that sex hormones and other sex-related factors may significantly influence the development and progression of osteosarcopenia. Further research using this animal model is needed to explore the role of sex hormones and other potential factors, such as inflammation and gut microbiota dysregulation (Picca et al., 2020) in the onset and progression of osteosarcopenia during aging.

Due to its unique characteristics, *O. degus* tuned out to be a valuable natural model of sex and age-related diseases or health complications (Cuenca-Bermejo et al., 2020), as demonstrated in several studies by this group and others on neurodegenerative diseases and synaptic dysfunctions (Tarragon et al., 2013; Oliva et al., 2022; Cuenca-Bermejo et al., 2021), cardiac function and the incidence of arrhythmias (Cuenca-Bermejo et al., 2023), working and spatial memory (Tarragon et al., 2014; Estrada et al., 2015a, 2015b; Estrada et al., 2019), as well as ocular alterations and neurochemical processing of vertical information (Chang et al., 2021; Szabadfi et al., 2015), hormonal dysfunction (Bustos Obregon and Ramirez, 1997), and onset of tumors and non-neoplastic proliferative lesions (Švara et al., 2020). To our knowledge, despite several works on preclinical rodent models (mice, rats) for human bone diseases (Koh et al., 2024), this foundational study is the first addressing the issue of osteosarcopenia in aging *O. degus*. Bases on these first results, *O. degus* also emerges as a promising candidate also for evaluating potential treatments and exploring genetic interventions designed for age-related musculoskeletal disorders. The search for potential biomarkers in the gerontology is ongoing with promising findings for new diagnostic markers (Albright et al., 1941; Calvani et al., 2020). For instance, levels of two inflammatory mediators - platelet-derived growth factor BB, which is crucial for skeletal muscle regeneration through the stimulation of satellite cells and myoblasts (Scully et al., 2018; Scully et al., 2019), and neutrophil-derived myeloperoxidase-are significantly decreased in the serum of patients with physical frailty and sarcopenia, indicating immunosenescence and reduced muscle regenerative capacity (Calvani et al., 2020). In these patients, increased levels of certain amino acids (Kouchiwa et al., 2012), such as aspartic acid and asparagine (metabolic intermediates for glutamine synthesis), and citrulline (synthetized from glutamine), suggest deregulation of nitrogen and glutamine metabolism in muscles (Calvani et al., 2020). Similarly, reduced levels of α-aminobutyric acid, indicative of deregulation of glutathione synthesis and redox balance, along with a reduction in heat shock protein 72 - produced in response to different stressors (Johnson and Fleshner, 2006) and known to slow the progression of muscular dystrophy (Gehrig et al., 2012) while increasing muscle oxidative capacity (Henstridge et al., 2014) - indicate altered management of hormetic stress signals (Calvani et al., 2020). Additionally, the detailed study of Calvani et al. (2020) has highlighted gender-specific differences in biomarker profiles between patients with physical frailty and sarcopenia and elderly individuals without these pathological conditions, which aligns with the sex-related differences identified in our study.

Vitamin D levels are a critical marker for osteoporosis, playing a key role in optimal bone growth and calcium-phosphate homeostasis (Chevalley et al., 2022). Additionally, oxidative stress markers -such as elevated levels of malondialdehyde, advanced oxidation protein products and vitamin B12, along with reduced antioxidants like catalase, glutathione peroxidase, uric acid and folate-have been significantly associated with osteoporosis (Zhao et al., 2021). Consistent with these findings, *O. degus* shows promise as a candidate for further research on osteosarcopenia biomarkers.

In a study conducted in the Senescence Accelerated Mouse-Prone 8 (SAMP8) model, a naturally occurring mouse line exhibiting accelerated aging, a clear link was demonstrated between sarcopenia and impaired fracture recovery due to osteoporosis (Zhang et al., 2017). Our findings, which reveal a significant reduction in muscle mass and bone mineral density in the hindlimbs of old and senile *O. degus* of both sexes, suggest an increased risk of fractures (Cefalu, 2004) and associated diseases like cardiovascular conditions (Barbalho et al., 2020). Moreover, the greater loss of muscle and bone in the hindlimbs compared to the forelimbs in this model mirrors disease progression in humans, where most fractures occur in the lower limbs, including the pelvic region (Oberkircher et al., 2018).

In conclusion, the demographic shift toward an aging population in developed countries underscore the need for a reliable animal model to study the effects of aging and identify early indicators of age-related diseases such as osteosarcopenia. The impact of sarcopenia on the development of other diseases has been well-documented in numerous studies (Moorthi and Avin, 2017; Bone et al., 2017; Fukushima et al., 2018; Kim et al., 2019). Because its characteristics, *O. degus* presents a valuable opportunity to advance research in this field, offering insights into the underlying mechanisms of osteosarcopenia and its potential links to other diseases (Cuenca-Bermejo et al., 2020). This primarily relies on the lifespan of *O. degus*, which, in captivity, can extend for several years. This longevity allows for longitudinal studies that track the gradual progression of aging, enabling a more precise characterization of the onset and development of age-related diseases and their potential comorbidities. Additionally, integrating neurological and musculoskeletal metrics facilitates the exploration of the interplay between neurodegeneration and musculoskeletal decline, which share common underlying factors such as inflammation, oxidative stress, and mitochondrial dysfunction. Moreover, this type of research can be conducted in a controlled environment, where the confounding effects of diet, climate and physical exercise on the aging process (Li et al., 2019; Kobyliansky et al., 2000) are minimized. Such studies are crucial for developing targeted interventions and improving healthcare outcomes in an aging population. Finally, with ageing, sex differences were also observed, with female *O. degus* having greater cortical bone volume in both hind and forelimbs, and greater muscle mass in the sole hindlimbs, compared to male. These findings enhance the characterization of *O. degus* as a model also do delve into to sex-related differences in aging pathologies.

We acknowledge that while CT, along with Magnetic Resonance Imaging, are valuable research tools, techniques such as Dual-energy X-ray Absorptiometry (DXA) are predominantly used in clinical settings for diagnosing osteoporosis and sarcopenia in humans. DXA is widely employed for measuring both bone mineral density and muscle mass, often in combination with functional tests such as grip strength measurements and physical performance assessments (Messina et al., 2018). However, it is worth emphasizing that CT is increasingly being utilized in clinical practice and, alongside MRI, is considered the gold standard for evaluating body composition. CT provides precise measurements of muscle volume and enables the calculation of total muscle mass, making it a more viable option for clinical use, particularly in cases where detailed imaging is required or when DXA results are inconclusive (Messina et al., 2018). Like any technique, the use of CT imaging has its limitations (Chianca et al., 2022) and does not always allow for more conclusive statements on the aging model *O. degus* beyond those highlighted previously. Nevertheless, this study establishes a solid foundation of knowledge to support future, more dynamic investigations. These could include analyses of markers reflecting the dynamic nature of muscle and bone metabolism (Al Saedi et al., 2019; Al Saedi et al., 2024), as well as the biological processes involved in osteosarcopenia.

## Data Availability

The datasets presented in this study can be found in online repositories. The names of the repository/repositories and accession number(s) can be found below: 10.6084/m9.figshare.26781322.

## Supplementary material

SUPPLEMETARY FIGURE 1 Representative computerized tomography images of male and female hindlimbs, including the femur and biceps femoris, at different life stages: juvenile, adult, old, and senile. Each image is presented in its original form and with the region of interest (ROI), highlighted in red, where analyses were conducted. Scale bar: 5 mm.

## Abbreviations

BMD, Bone mineral density; CT, Computed tomography; LSO, Lutetium orthosilicates cristal; MRI, Magnetic resonance imaging; MIP-1β, Macrophage inflammatory protein *O. degus*, *Octodon degus*; PDGF-BB, Platelet-derived growth factor BB; ROI, Region of interest; SD, Standard deviation; VOI, Volume of intere.

## References

[B1] Albright F. Smith P. H. Richardson A. M. (1941). Postmenopausal osteoporosis: its clinical features. JAMA 116 (22), 2465–2474. 10.1001/jama.1941.02820220007002

[B2] Al Saedi A. Feehan J. Phu S. Duque G. (2019). Current and emerging biomarkers of frailty in the elderly. Clin. Interv. Aging 14, 389–398. 10.2147/CIA.S168687 30863033 PMC6388773

[B3] Al Saedi A. Yacoub A. s. Awad K. Karasik D. Brotto M. Duque G. (2024). The interplay of lipid signaling in musculoskeletal cross talk: implications for health and disease. Methods Mol. Biol. 2816, 1–11. 10.1007/978-1-0716-3902-3_1 38977583

[B4] Arron J. R. Choi Y. (2000). Bone versus immune system. Nature 408 (6812), 535–536. 10.1038/35046196 11117729

[B5] Axell A. M. Maclean D. R. Plant L. J. Harcourt L. J. Davis J. A. Jimenez M. (2006). Continuous testosterone administration prevents skeletal muscle atrophy and enhances resistance to fatigue in orchidectomized male mice. Am. J. Physiol. Endocrinol. Metab. 291, E506–E516. 10.1152/ajpendo.00058.2006 16621900

[B6] Baltgalvis K. A. Greising S. M. Warren G. L. Lowe D. A. (2010). Estrogen regulates estrogen receptors and antioxidant gene expression in mouse skeletal muscle. PLoS ONE 5 (4), e10164. 10.1371/journal.pone.0010164 20405008 PMC2854140

[B7] Barak M. M. (2024). Cortical and trabecular bone modeling and implications for bone functional adaptation in the mammalian tibia. Bioengineering 11, 514. 10.3390/bioengineering11050514 38790379 PMC11118124

[B8] Barbalho S. M. Flato U. A. P. Tofano R. J. Goulart R. D. A. Guiguer E. L. Detregiachi C. R. P. (2020). Physical exercise and myokines: relationships with sarcopenia and cardiovascular Complications. Int. J. Mol. Sci. 21 (10), 3607. 10.3390/ijms21103607 32443765 PMC7279354

[B9] Barwick A. Tessier J. Mirow J. de Jonge X. J. Chuter V. (2017). Computed tomography derived bone density measurement in the diabetic foot. J. Foot Ankle Res. 10, 11. 10.1186/s13047-017-0192-7 28270861 PMC5335776

[B10] Bhasin S. Storer T. W. Berman N. Callegari C. Clevenger B. Phillips J. (1996). The effects of supraphysiologic doses of testosterone on muscle size and strength in normal men. N. Engl. J. Med. 335, 1–7. 10.1056/NEJM199607043350101 8637535

[B11] Bone A. E. Hepgul N. Kon S. Maddocks M. (2017). Sarcopenia and frailty in chronic respiratory disease. Chron. Respir. Dis. 14 (1), 85–99. 10.1177/1479972316679664 27923981 PMC5720213

[B12] Britton R. A. Irwin R. Quach D. Schaefer L. Zhang J. Lee T. (2014). Probiotic L. reuteri treatment prevents bone loss in a menopausal ovariectomized mouse model. J. Cell Physiol. 229 (11), 1822–1830. 10.1002/jcp.24636 24677054 PMC4129456

[B13] Bustos Obregon E. Ramirez O. (1997). Ageing and testicular function in *Octodon degus* . Andrologia 29, 319–326. 10.1111/j.1439-0272.1997.tb00325.x 9430437

[B14] Calvani R. Picca A. Marini F. Biancolillo A. Gervasoni J. Persichilli S. (2020). Identification of biomarkers for physical frailty and sarcopenia through a new multi-marker approach: results from the BIOSPHERE study. GeroScience 43 (2), 727–740. 10.1007/s11357-020-00197-x 32488674 PMC8110636

[B15] Cefalu C. A. (2004). Is bone mineral density predictive of fracture risk reduction? Curr. Med. Res. Opin. 20 (3), 341–349. 10.1185/030079903125003062 15025843

[B16] Chang Y.-L. Palanca-Castan N. Neira D. Palacios A. G. Acosta M. L. (2021). Ocular health of *Octodon degus* as a clinical marker for age-related and age-independent neurodegeneration. Front. Integr. Neurosci. 15, 665467. 10.3389/fnint.2021.665467 33927598 PMC8076605

[B17] Chevalley T. Brandi M. L. Cashman K. D. Cavalier E. Harvey N. C. Maggi S. (2022). Role of vitamin D supplementation in the management of musculoskeletal diseases: update from an European society of clinical and economical aspects of osteoporosis, osteoarthritis and musculoskeletal diseases (ESCEO) working group. Aging Clin. Exp. Res. 34 (11), 2603–2623. 10.1007/s40520-022-02279-6 36287325 PMC9607746

[B18] Chianca V. Albano D. Messina C. Gitto S. Ruffo G. Guarino S. (2022). Sarcopenia: imaging assessment and clinical application. Abdom. Radiol. (NY) 47, 3205–3216. 10.1007/s00261-021-03294-3 34687326 PMC8536908

[B19] Christian C. J. Benian G. M. (2020). Animal models of sarcopenia. Aging Cell 19 (10), e13223. 10.1111/acel.13223 32857472 PMC7576270

[B20] Clynes M. A. Gregson C. L. Bruyère O. Cooper C. Dennison E. M. (2021). Osteosarcopenia: where osteoporosis and sarcopenia collide. Rheumatology 60 (2), 529–537. 10.1093/rheumatology/keaa755 33276373

[B21] Clynes M. A. Harvey N. C.-Curtis E. M. Fuggle N. R.-Dennison E. M. Cooper C. (2020). The epidemiology of osteoporosis. Br. Med. Bull. 133 (1), 105–117. 10.1093/bmb/ldaa005 32282039 PMC7115830

[B22] Cruz-Jentoft A. J. Bahat G. Bauer J. Boirie Y. Bruyère O. Cederholm T. (2019). Sarcopenia: revised European consensus on definition and diagnosis. Age Ageing 48 (1), 601–631. 10.1093/ageing/afz046 PMC6593317 31081853

[B23] Cuenca-Bermejo L. Fernández-Del Palacio M. J. de Cassia Gonçalves V. Bautista-Hernández V. Sánchez-Rodrigo C. Fernández-Villalba E. (2023). Age and sex determine electrocardiogram parameters in the *Octodon degus* . Biology 12, 747. 10.3390/biology12050747 37237559 PMC10215068

[B24] Cuenca-Bermejo L. Pizzichini E. Gonçalves V. C. Guillén-Díaz M. Aguilar-Moñino E. Sánchez- Rodrigo C. (2021). A new tool to study parkinsonism in the context of aging: MPTP intoxication in a natural model of multimorbidity. Int. J. Mol. Sci. 22, 4341. 10.3390/ijms22094341 33919373 PMC8122583

[B25] Cuenca-Bermejo L. Pizzichini E. Gonzalez-Cuello A. M.-De Stefano M. E. Fernandez-Villalba E. Herrero M.-T. (2020). *Octodon degus*: a natural model of multimorbidity for ageing research. Ageing Res. Rev. 64, 101204. 10.1016/j.arr.2020.101204 33152453

[B26] de Lucena Alves C. P. Câmara M. Dantas Macêdo G. A. Freire Y. A. de Melo Silva R. Paulo-Pereira R. (2022a). Agreement between upper and lower limb measures to identify older adults with low skeletal muscle strength, muscle mass and muscle quality. PLoS ONE 17 (1), e0262732. 10.1371/journal.pone.0262732 35061817 PMC8782376

[B27] de Lucena Alves C. P. de Almeida S. B. Pessoa Lima D. Braga Neto P. Miranda A. L. Manini T. (2022b). Muscle quality in older adults: a scoping review. J. Am. Med. Dir. Ass. 24 (4), 462–467e12. 10.1016/J.jamda.2023.02.012 36963436

[B28] Elissamburu A. De Santis L. (2011). Forelimbs proportions and fossoria adaptation in the scratch-digging rodent *Ctenomys* (Caviomorpha). J. Mammal. 92 (3), 683–689. 10.1644/09-MAMM-A-113.1

[B29] Estrada C. Cuenca-Bermejo L. Cano-Fernandez L. Gil-Martinez A. L. Sanchez-Rodrigo C. González-Cuello A. M. (2019). Voluntary exercise reduces plasma cortisol levels and improves transitory memory impairment in young and aged *Octodon degus* . Behav. Brain Res. 373, 112066. 10.1016/j.bbr.2019.112066 31269420

[B30] Estrada C. Fernández-Gómez F. J. López D. Gonzalez-Cuello A. Tunez I. Toledo F. (2015a). Transcranial magnetic stimulation and aging: effects on spatial learning and memory after sleep deprivation in *Octodon degus* . Neurobiol. Learn. Mem. 125, 274–281. 10.1016/j.nlm.2015.09.011 26463507

[B31] Estrada C. López D. Conesa A. Fernández-Gómez F. J. Gonzalez-Cuello A. Toledo F. (2015b). Cognitive impairment after sleep deprivation rescued by transcranial magnetic stimulation application in *Octodon degus* . Neurotox. Res. 28 (4), 361–371. 10.1007/s12640-015-9544-x 26194615

[B32] Ethgen O. Beaudart C. Buckinx F. Bruyère O. Reginster J. Y. (2017). The future prevalence of sarcopenia in Europe: a claim for public health action. Calcif. Tissue Int. 100 (3), 229–234. 10.1007/s00223-016-0220-9 28012107 PMC5313588

[B33] Fagundes Belchior G. Kirk B. Da Pereira Silva E. A. Duque G. (2020). Osteosarcopenia: beyond age-related muscle and bone loss. Eur. Geriatr. Med. 11 (5), 715–724. 10.1007/s41999-020-00355-6 32676865

[B34] Farmer R. E. Mathur R. Schmidt A. F. Bhaskaran K. Fatemifar G. Eastwood S. V. (2019). Associations between measures of sarcopenic obesity and risk of cardiovascular disease and mortality: a cohort study and mendelian randomization analysis using the UK biobank. J. Am. Heart Assoc. 8 (13), e011638. 10.1161/JAHA.118.011638 31221000 PMC6662360

[B35] Figueiredo C. P. Kleyer A. Simon D. Stemmler F. d’Oliveira I. Weissenfels A. (2018). Methods for segmentation of rheumatoid arthritis bone erosions in high-resolution peripheral quantitative computed tomography (HR-pQCT). Sem. Arthrit. Rheumat. 47 (5), 611–618. 10.1016/j.semarthrit.2017.09.011 29122245

[B36] Frontera W. R. Hughes V. A. Lutz K. J. Evans W. J. (1991). A cross-sectional study of muscle strength and mass in 45- to 78-yr-old men and women. J. Appl. Physiol. 71 (2), 644–650. 10.1152/jappl.1991.71.2.644 1938738

[B37] Fukushima H. Takemura K. Suzuki H. Koga F. (2018). Impact of sarcopenia as a prognostic biomarker of bladder cancer. Int. J. Mol. Sci. 19 (10), 2999. 10.3390/ijms19102999 30275370 PMC6213561

[B38] Gehrig S. M. van der Poel C. Sayer T. A. Schertzer J. D. Henstridge D. C. Church J. E. (2012). Hsp72 preserves muscle function and slows progression of severe muscular dystrophy. Nature 484 (7394), 394–398. 10.1038/nature10980 22495301

[B95] Hassan E. B. Duque G. (2017). Osteosarcopenia: A new geriatric syndrome. Aust. Fam. Physician 46, 894–853. 29101922

[B39] He H. Liu Y. Tian Q. Papasian C. J. Hu T. Deng H. W. (2016). Relationship of sarcopenia and body composition with osteoporosis. Osteoporos. Int. 27, 473–482. 10.1007/s00198-015-3241-8 26243357

[B40] Henstridge D. C. Bruce C. R. Drew B. G. Tory K. Kolonics A. Estevez E. (2014). Activating HSP72 in rodent skeletal muscle increases mitochondrial number and oxidative capacity and decreases insulin resistance. Diabetes 63 (6), 1881–1894. 10.2337/db13-0967 24430435 PMC4030108

[B41] Hernlund E. Svedbom A. Ivergård M. Compston J. Cooper C. Stenmark J. (2013). Osteoporosis in the European union: medical management, epidemiology and economic burden: a report prepared in collaboration with the international osteoporosis foundation (IOF) and the European federation of pharmaceutical industry associations (EFPIA). Arch. Osteoporos. 8 (1), 136. 10.1007/s11657-013-0136-1 24113837 PMC3880487

[B42] Hirschfeld H. P. Kinsella R. Duque G. (2017). Osteosarcopenia: where bone, muscle, and fat collide. Osteoporos. Int. 28 (10), 2781–2790. 10.1007/s00198-017-4151-8 28733716

[B43] Hosoi T. Yakabea M. Sasakawa H. Sasako T. Ueki K. Kato S. (2023). Sarcopenia phenotype and impaired muscle function in male mice with fast-twitch muscle-specific knockout of the androgen receptor. PNAS 120 (4), e2218032120. 10.1073/pnas.2218032120 36669097 PMC9942915

[B44] Inestrosa N. C. Reyes A. E. Chacón M. A. Cerpa W. Villalón A. Montiel J. (2005). Human-like rodent amyloid-beta-peptide determines Alzheimer pathology in aged wild-type Octodon degu. Neurobiol. Aging 26 (7), 1023–1028. 10.1016/j.neurobiolaging.2004.09.016 15748782

[B45] Johnson J. D. Fleshner M. (2006). Releasing signals, secretory pathways, and immune function of endogenous extracellular heat shock protein 72. J. Leukoc. Bio. 79 (3), 425–434. 10.1189/jlb.0905523 16387837

[B46] Kenkre J. S. Bassett J. (2018). The bone remodelling cycle. Ann. Clin. Biochem. 55 (3), 308–327. 10.1177/0004563218759371 29368538

[B96] Kim Y. M. Kim J. -H. Baik S. J. Chun J. Youn Y. H. Park H. (2019). Sarcopenia and sarcopenic obesity as novel risk factors for gastric carcinogenesis: A health checkup cohort study. Front. Oncol. 9, 1249. 10.3389/fonc.2019.01249 31799199 PMC6868021

[B47] Kirk B. Al Saedi A. (2019). Osteosarcopenia: a case of geroscience. Aging Med. 2 (3), 147–156. 10.1002/agm2.12080 PMC6880711 31942528

[B48] Kirk B. Zanker J. Duque G. (2020). Osteosarcopenia: epidemiology, diagnosis, and treatment-facts and numbers. J. Cachexia Sarcopenia Muscle 11 (3), 609–618. 10.1002/jcsm.12567 32202056 PMC7296259

[B49] Klibanski A. Adams-Campbell L. Bassford T. Blair S. N. Boden S. D. Dickersin K. (2001). Osteoporosis prevention, diagnosis, and therapy. JAMA 285 (6), 785–795. 10.1001/jama.285.6.785 11176917

[B50] Ko Y. Shin Y. Sung Y. S. Lee J. Lee J. H. Kim J. K. (2022). A reliable and robust method for the upper thigh muscle quantification on computed tomography: toward a quantitative biomarker for sarcopenia. BMC Musculoskelet. Dis. 23 (1), 93. 10.1186/s12891-022-05032-2 PMC8796642 35086521

[B51] Kobyliansky E. Karasik D. Belkin V. Livshits G. (2000). Bone ageing: genetics versus environment. Ann. Hum. Biol. 27 (5), 433–451. 10.1080/030144600419288 11023115

[B52] Koh N. Y. Y. Miszkiewicz J. J. Fac M. L. Wee N. K. Y. Sims N. A. (2024). Preclinical rodent models for human bone disease, including a focus on cortical bone. End. Rev. 45, 493–520. 10.1210/endrev/bnae004 PMC11244217 38315213

[B53] Komori T. (2015). Animal models for osteoporosis. Eur. J. Pharmacol. 759, 287–294. 10.1016/j.ejphar.2015.03.028 25814262

[B54] Kouchiwa T. Wada K. Uchiyama M. Kasezawa N. Niisato M. Murakami H. (2012). Age-related changes in serum amino acids concentrations in healthy individuals. Clin. Chem. Lab. Med. 50 (5), 861–870. 10.1515/cclm-2011-0846 22628330

[B55] Krut’ko V. N. Dontsov V. I. Khalyavkin A. V. Markova A. M. (2018). Natural aging as as a sequential poly-systemic syndrome. Front. Biosci. 23 (3), 909–920. 10.2741/4624 28930580

[B56] Labyak S. E. Lee T. M. (1995). Estrus- and steroid-induced changes in circadian rhythms in a diurnal rodent, *Octodon degus* . Octodon Degus. Physiol. Behav. 58, 573–585. 10.1016/0031-9384(95)00096-2 8587967

[B57] Lang T. Streeper T. Cawthon P. Baldwin K. Taaffe D. R. Harris T. B. (2010). Sarcopenia: etiology, clinical consequences, intervention, and assessment. Osteoporos. Int. 21, 543–559. 10.1007/s00198-009-1059-y 19779761 PMC2832869

[B58] Leser J. M. Harriot A. Buck H. V. Ward C. W. Stains J. P. (2021). Aging, osteo-sarcopenia, and musculoskeletal mechano-transduction. Front. Rehabilit. Sci. 2, 782848. 10.3389/fresc.2021.782848 PMC9396756 36004321

[B59] Li G. B. Zhang L. Wang D. E. Qudsy L. A. I. Jiang J. X. Xu H. Y. (2019). Muscle‐bone crosstalk and potential therapies for sarco‐osteoporosis. J. Cell. Biochem. 120 (9), 14262–14273. 10.1002/jcb.28946 31106446 PMC7331460

[B60] Liguori I. Russo G. Aran L. Bulli G. Curcio F. Della-Morte D. (2018). Sarcopenia: assessment of disease burden and strategies to improve outcomes. Clin. Interv. Aging 13, 913–927. 10.2147/CIA.S149232 29785098 PMC5957062

[B61] Lynch N. A. Metter E. J. Lindle R. S. Fozard J. L. Tobin J. D. Roy T. A. (1999). Muscle quality. I. Age-associated differences between arm and leg muscle groups. J. Appl. Physiol. 86 (1), 188–194. 10.1152/jappl.1999.86.1.188 9887130

[B62] MacLean H. E. Chiu W. S. M. Notini A. J. Axell A.-M. Davey R. A. McManus J. F. (2008). Impaired skeletal muscle development and function in male, but not female, genomic androgen receptor knockout mice. Faseb J. 22 (8), 2676–2689. 10.1096/fJ.08-105726 18390925

[B63] Mahoney M. M. Rossi B. V. Hagenauer M. H. Lee T. M. (2011). Characterization of the estrous cycle in *Octodon degus* . Biol. Reprod. 84, 664–671. 10.1095/biolreprod.110.087403 21084711 PMC3062035

[B64] Maravilla K. R. Murry R. C. Jr. (1981). Computed tomography: basic principles of operation. Clin. Neurosurg. 28, 482–501. 10.1093/neurosurgery/28.cn_suppl_1.482 7318296

[B65] Melton L. J. (2001). The prevalence of osteoporosis: gender and racial comparison. Calcif. Tissue Int. 69 (4), 179–181. 10.1007/s00223-001-1043-9 11730244

[B66] Messina C. Maffi G. Vitale J. A. Ulivieri F. M. Guglielmi G. Sconfienza L. M. (2018). Diagnostic imaging of osteoporosis and sarcopenia: a narrative review. Quant. Imaging Med. Surg. 8, 86–99. 10.21037/qims.2018.01.01 29541625 PMC5835659

[B67] Metter E. J. Lynch N. Conwit R. Lindle R. Tobin J. Hurley B. (1999). Muscle quality and age: cross-sectional and longitudinal comparisons. J. Gerontol. A Biol. Sci. Med. Sci. 54 (5), B207–B218. 10.1093/gerona/54.5.b207 10362000

[B68] Moorthi R. N. Avin K. G. (2017). Clinical relevance of sarcopenia in chronic kidney disease. Curr. Opin. Nephrol. Hypertens. 26 (3), 219–228. 10.1097/MNH.0000000000000318 28198733 PMC5860815

[B69] Morgan E. F. Unnikrisnan G. U. Hussein A. I. (2018). Bone mechanical properties in healthy and diseased states. Annu. Rev. Biomed. Eng. 20, 119–143. 10.1146/annurev-bioeng-062117-121139 29865872 PMC6053074

[B70] Oberkircher L. Ruchholtz S. Rommens P. M. Hofmann A. Bücking B. Krüger A. (2018). Osteoporotic pelvic fractures. Dtsch. Arztebl. Int. 115 (5), 70–80. 10.3238/arztebl.2018.0070 29439771 PMC5817189

[B71] Oheim R. Schinke T. Amling M. Pogoda P. (2016). Can we induce osteoporosis in animals comparable to the human situation? Inj. Suppl. 1, S3–S9. 10.1016/S0020-1383(16)30002-X 26768287

[B72] Ohlsson C. Engdahl C. Fåk F. Andersson A. Windahl S. H. Farman H. H. (2014). Probiotics protect mice from ovariectomy-induced cortical bone loss. PloS One 9 (3), e92368. 10.1371/journal.pone.0092368 24637895 PMC3956931

[B73] Oliva C. A. Rivera D. S. Mariqueo T. A. Bozinovic F. Inestrosa N. C. (2022). Differential role of sex and age in the synaptic transmission of degus (*Octodon degus*). Front. Integr. Neurosci. 16, 799147. 10.3389/fnint.2022.799147 35295186 PMC8918727

[B74] Pascual-Fernández J. Fernández-Montero A. Córdova-Martínez A. Pastor D. Martínez-Rodríguez A. Roche E. (2020). Sarcopenia: molecular pathways and potential targets for Intervention. Int. J. Mol. Sci. 21 (22), 8844. 10.3390/ijms21228844 33266508 PMC7700275

[B75] Pearson O. M. Lieberman D. E. (2004). The aging of Wolff’s “Law”: ontogeny and responses to mechanical loading in cortical bone. Yearb. Physic. Antropol. 47, 63–99. 10.1002/ajpa.20155 15605390

[B76] Picca A. M. Ponziani F. R. Calvani R. Marini F. Biancolillo A. CoelhoJúnior H. J. (2020). Gut microbial, inflammatory and metabolic signatures in older people with physical frailty and sarcopenia: results from the BIOSPHERE study. Nutrients 12 (1), 65. 10.3390/nu12010065 PMC7019826 31887978

[B77] Richelson L. S. Wahner H. W. Melton III L. J. Riggs B. L. (1984). Relative contributions of aging and estrogen deficiency to postmenopausal bone loss. N. Engl. J. Med. 311 (20), 1273–1275. 10.1056/NEJM198411153112002 6493283

[B78] Scully D. Naseem K. M. Matsakas A. (2018). Platelet biology in regenerative medicine of skeletal muscle. Acta Physiol. 223 (3), e13071. 10.1111/apha.13071 29633517

[B79] Scully D. Sfyri P. Verpoorten S. Papadopoulos P. Muñoz-Turrillas M. C. Mitchell R. (2019). Platelet releasate promotes skeletal myogenesis by increasing muscle stem cell commitment to differentiation and accelerates muscle regeneration following acute injury. Acta Physiol. 225 (3), e13207. 10.1111/apha.13207 30339324

[B80] Sharples A. P. Hughes D. C. Deane C. S. Saini A. Selman C. Stewart C. E. (2015). Longevity and skeletal muscle mass: the role of IGF signalling, the sirtuins, dietary restriction and protein intake. Aging Cell 14 (4), 511–523. 10.1111/acel.12342 25866088 PMC4531066

[B81] Singer A. (2006). Osteoporosis diagnosis and screening. Clin. Cornerstone 8 (1), 9–18. 10.1016/s1098-3597(06)80061-x 17591572

[B82] Švara T. Gombaˇc M. Poli A. Raˇcnik J. Zadravec M. (2020). Spontaneous tumor and non-neoplastic proliferative lesions in pet Degus (*Octodon Degus*). Vet. Sci. 7, 32. 10.3390/vetsci7010032 32183187 PMC7158670

[B83] Szabadfi K. Estrada C. Fernandez-Villalba E. Tarragon E. Setalo G. Jr Izura V. (2015). Retinal aging in the diurnal Chilean rodent (*Octodon degus*): histological, ultrastructural and neurochemical alterations of the vertical information processing pathway. Front. Cell. Neurosci. 9, 126. 10.3389/fncel.2015.00126 25954153 PMC4405622

[B84] Talbot J. Maves L. (2016). Skeletal muscle fiber type: using insights from muscle developmental biology to dissect targets for susceptibility and resistance to muscle disease. WIREs Dev. Biol. 5, 518–534. 10.1002/wdev.230 PMC5180455 27199166

[B85] Tarragon E. Lopez D. Estrada C. Ana G. C. Schenker E. Pifferi F. (2013). *Octodon degus*: a model for the cognitive impairment associated with alzheimer's disease. CNS Neurosci. Ther. 19 (9), 643–648. 10.1111/cns.12125 23710760 PMC6493546

[B86] Tarragon E. Lopez D. Estrada C. Gonzalez-Cuello A. Ros C. M. Lamberty Y. (2014). Memantine prevents reference and working memory impairment caused by sleep deprivation in both young and aged *Octodon degus* . Neuropharmacology 85, 206–214. 10.1016/j.neuropharm.2014.05.023 24878242

[B87] Tournadre A. Vial G. Capel F. Soubrier M. Boirie Y. (2019). Sarcopenia. Jt. Bone Spine 86 (3), 309–314. 10.1016/j.jbspin.2018.08.001 30098424

[B89] Wang C. Swerdloff R. S. Iranmanesh A. Dobs A. Snyder P. J. Cunningham G. (2000). Transdermal testosterone gel improves sexual function, mood, muscle strength, and body composition parameters in hypogonadal men. J. Clin. Endocrinol. Metab. 85, 2839–2853. 10.1210/jcem.85.8.6747 10946892

[B90] Wozniak A. C. Anderson J. E. (2009). The dynamics of the nitric oxide release-transient from stretched muscle cells. Int. J. Biochem. Cell Biol. 41 (3), 625–631. 10.1016/j.biocel.2008.07.005 18694846

[B91] Wu D. Cline-Smith A. Shashkova E. Perla A. Katyal A. Aurora R. (2021). T-Cell Mediated inflammation in postmenopausal osteoporosis. Front. Immunol. 12, 687551. 10.3389/fimmu.2021.687551 34276675 PMC8278518

[B92] Yoh K. Ikeda K. Horie K. Inoue S. (2023). Roles of estrogen, estrogen receptors, and estrogen-related receptors in skeletal muscle: regulation of mitochondrial function. Int. J. Mol. Sci. 24, 1853. 10.3390/ijms24031853 36768177 PMC9916347

[B93] Zhang N. Chow S. K. H. Leung K. S. Lee H. H. Cheung W. H. (2017). An animal model of co-existing sarcopenia and osteoporotic fracture in senescence accelerated mouse prone 8 (SAMP8). Exp. Gerontol. 97, 1–8. 10.1016/j.exger.2017.07.008 28711604

[B94] Zhao F. Guo L. Wang X. Zhang Y. (2021). Correlation of oxidative stress-related biomarkers with postmenopausal osteoporosis: a systematic review and meta-analysis. Arch. Osteopor. 16 (1), 4. 10.1007/s11657-020-00854-w 33400044

