# Correction: Age- and sex-related development of osteosarcopenia in the aging *Octodon degus* rodent model

**DOI:** 10.3389/fragi.2025.1669031

**Published:** 2025-07-31

**Authors:** Pablo Gallo-Soljancic, Maria Egle De Stefano, Ana-Maria Lucas-Ochoa, Consuelo Sánchez-Rodrigo, Lorena Cuenca-Bermejo, Ana-María González-Cuello, Emiliano Fernández-Villalba, Maria-Trinidad Herrero

**Affiliations:** ^1^ Clinical & Experimental Neuroscience (NiCE), Department of Human Anatomy and Psychobiology, Institute for Aging Research, School of Medicine, European University for Wellbeing (EUniWell), Campus Mare Nostrum, University of Murcia, Murcia, Spain; ^2^ Institute for Bio-Health Research of Murcia (IMIBPascual Parrilla), Campus of Health Sciences, El PalmarMurcia, Spain; ^3^ Department of Biology and Biotechnologies “Charles Darwin”, Sapienza University of Rome, Rome, Italy; ^4^ Center for research in Neurobiology “Daniel Bovet”, Sapienza University of Rome, Rome, Italy

**Keywords:** aging, osteoporosis, sarcopenia, Octodon degus, osteosarcopenia, sex

In the published article, an **Author name** was incorrectly written as Lorena Cuemca-Bermejo. The correct spelling is “Lorena Cuenca-Bermejo.”

In the published article, in the **Author contribution section**, the initials of one author was incorrectly written as MD. The correct initial is “MEDS.”

The original version of this article has been updated.

